# Supplementary material for: Performance of an Emergency Road Ambulance Service in Bhutan: Response Time, Utilization, and Outcomes
Source: Trop Med Infect Dis. 2022 May 31;7(6):87. doi: 10.3390/tropicalmed7060087 (PMC9227530; doi:10.3390/tropicalmed7060087)
Supplement: Supplementary file 1 [file tropicalmed-07-00087-s001.zip › tropicalmed-1739169-supplementary.pdf]

Health Help Center – Emergency Response Service

Ambulance Trip Sheet

(Fill up only for emergency ambulance services and not for inter-facility transfer)

Hospital Name: **Ambulance registration No:**
Event Date: **Event ID:**

**Ambulance Trip Details** (To be filled by drivers)		**Call details** (To be filled by Agents)	
Ambulance assigned time		Time of call	
Ambulance departure time		Name of caller	
Scene arrival time		Call location	
Scene departure time		Caller mobile no	
Hospital arrival time		Relation of caller to patient (please circle)	Parent/sibling/relative/friend/ neighbor/ office colleague /stranger (passerby)
Ambulance release time		**Patient demography and clinical details** (To be filled by Agent, Escort, EMR accompanying ambulance)	
Total distance travelled		Patient Name	
		CID/mobile no.	
**Ambulance service outcome (tick)** (To be filled by Driver/Escort/EMR)		Age/Sex	
		Occupation	
1. Transported safely and patient alive<br>2. Died before ambulance reached scene<br>3. Died on the way to hospital<br>4. Died on arrival at hospital<br>5. For pregnancy:<br>5.1 Safely reached hospital<br>5.2 Delivered before ambulance reached scene<br>5.3 Delivered in the ambulance<br>6. Ambulance transfer not required/refused after ambulance arrived at the scene		Diagnosis/Reason for the call	1. Medical case<br>2. Surgical case<br>3. Pregnancy related<br>4. Accident trauma<br>5. Road traffic accident<br>6. Others (specify)
		Patient status at scene	Critical/Severe/Moderate/Mild
		Medical care given at scene (briefly)	
**If there was an unusual/unexpected delay in ambulance deployment, please mention reasons here:			

- Every emergency call should fill this form for three months after from start date
- All Ambulance should send the filled form **every week** to 112 through scan copy in WhatsApp group OR email by Monday of the next week

1. **Ambulance driver (Name/Signature):**

2. **EMR/Escort (Name/Signature):**
